# Personal autonomy and hopelessness are associated with antidepressant drugs prescription in currently euthymic bipolar patients

**DOI:** 10.1192/j.eurpsy.2021.245

**Published:** 2021-08-13

**Authors:** G. Serafini, G. Vazquez, A. Aguglia, A. Amerio, M. Pompili, M. Amore

**Affiliations:** 1 Department Of Neuroscience, Rehabilitation, Ophthalmology, Genetics, Maternal And Child Health (dinogmi), University of Genoa, IRCCS Ospedale Policlinico San Martino, Genoa, Italy, Genoa, Italy; 2 International Consortium For Bipolar & Psychotic Disorders Research, McLean Hospital, Harvard Medical School, Boston, United States of America; 3 Neurosciences, Mental Health And Sensory Organs, Sapienza University of Rome, Rome, Italy; 4 Department Of Neuroscience, Rehabilitation, Ophthalmology, Genetics, Maternal And Child Health (dinogmi), Departimento di Neuroscienze, Università di Genova, Genoa, Italy

**Keywords:** Coping Strategies, Hopelessness, antidepressant medications, bipolar disorder

## Abstract

**Introduction:**

The patterns and clinical correlates related to antidepressant drugs (ADs) prescription for BD remain poorly understood.

**Objectives:**

This study aimed to compare socio-demographic and clinical features of BD patients treated vs. not treated with ADs.

**Methods:**

The sample consists of 287 currently euthymic bipolar patients. Among participants (mean age=51.9±15.02), 157 (40.1%) were receiving ADs.

**Results:**

Based on the main findings, subjects given ADs were older and more frequently retired than those without receiving ADs. Moreover, patients given ADs were more likely to have had a first major depressive episode and present with psychotic symptoms at illness onset. Lifetime substance abuse/dependence history was less frequently reported among patients given ADs. Furthermore, ADs given patients have a higher number of affective episodes, and longer duration of their illness. Additionally, subjects treated with ADs reported higher hopelessness levels, and lower positive reinterpretations than those who were not treated with ADs. Factors associated with ADs-use by multivariate modeling were reduced personal autonomy (OR=.070), and hopelessness levels (OR=1.391).

**Conclusions:**

These results may help clinicians to better understand the clinical correlates of BD subtypes and improve their differential management. Additional studies are needed to replicate these findings, and facilitate the differential trajectories of BD patients based on socio-demographic/clinical profile.

**Disclosure:**

No significant relationships.

